# C8orf76 Modulates Ferroptosis in Liver Cancer via Transcriptionally Up-Regulating SLC7A11

**DOI:** 10.3390/cancers14143410

**Published:** 2022-07-13

**Authors:** Duguang Li, Junhai Pan, Yiyin Zhang, Yirun Li, Shengxi Jin, Cheng Zhong, Peng Chen, Jingjing Ma, Wendi Hu, Xiaoxiao Fan, Hui Lin

**Affiliations:** 1 Department of General Surgery, Sir Run Run Shaw Hospital, School of Medicine, Zhejiang University, 3 East Qingchun Rd., Hangzhou 310016, China; 11918306@zju.edu.cn (D.L.); 3413017@zju.edu.cn (J.P.); yiyinzhang@zju.edu.cn (Y.Z.); 11818184@zju.edu.cn (Y.L.); 11918289@zju.edu.cn (S.J.); 11918318@zju.edu.cn (C.Z.); chenpeng0818@zju.edu.cn (P.C.); 2 Department of Plastic Surgery, Sir Run Run Shaw Hospital, School of Medicine, Zhejiang University, Hangzhou 310016, China; 3410030@zju.edu.cn; 3 Division of Hepatobiliary and Pancreatic Surgery, Department of Surgery, The First Affiliated Hospital, School of Medicine, Zhejiang University, Hangzhou 310016, China; huwendi@zju.edu.cn; 4 Zhejiang Engineering Research Center of Cognitive Healthcare, Sir Run Run Shaw Hospital, School of Medicine, Zhejiang University, Hangzhou 310016, China; 5 College of Biomedical Engineering and Instrument Science, Zhejiang University, Hangzhou 310016, China

**Keywords:** hepatocellular carcinoma, ferroptosis, C8orf76, SLC7A11, ferroptosis resistance, oxidative stress

## Abstract

**Simple Summary:**

Chromosome 8 open reading frame 76 (C8orf76), a novel gene located in the nucleus, is highly expressed in many tumor types. Here, we present novel insights into the molecular mechanism and function of C8orf76 in HCC via in vitro and in vivo assays. On the one hand, C8orf76 could play a vital role in cell proliferation and cell cycle progression. More importantly, on the other hand, C8orf76 also acts as an important regulator of ferroptosis in HCC through activating SLC7A11 transcriptionally, resulting in elevation of GSH synthesis and lipid peroxidation resistance. Our study indicated that C8orf76 could be a novel marker for HCC diagnosis and therapeutic target for HCC patients.

**Abstract:**

Hepatocellular carcinoma (HCC) is a common malignant tumor worldwide. Chromosome 8 open reading frame 76 (C8orf76), a novel gene located in the nucleus, is highly expressed in many tumor types. However, the specific mechanisms and functions of C8orf76 in HCC remain unclear. Here, we reported for the first time that C8orf76 gene expression levels were frequently upregulated in liver cancer and significantly correlated with HCC development. C8orf76 downregulation induced G1-S arrest and inhibited cell proliferation. Intriguingly, C8orf76 deficiency could accelerate erastin or sorafenib-induced ferroptosis through increasing lipid reactive oxygen species (ROS) levels. Moreover, although C8orf76 overexpression did not affect tumorigenesis under normal conditions, it increased resistance to lipid disturbance and ferroptosis triggered by erastin or sorafenib, which further facilitated HCC cell growth and tumor progression. Mechanistically, C8orf76 bound to the promoter region of the solute carrier family 7 member 11 (SLC7A11) gene and upregulated SLC7A11 transcriptionally. SLC7A11-dependent cystine import led to sufficient GSH synthesis and lipid peroxidation inhibition, thus accelerating tumor growth. Our study indicated that C8orf76 could be a novel marker for HCC diagnosis. In addition, a better comprehensive understanding of the potential role of C8orf76 in HCC helped us develop novel therapeutic strategies for this intractable cancer.

## 1. Introduction

Hepatocellular carcinoma (HCC) is one of the most prevalent tumors observed in the world and the third most lethal cancer with an oncological origin [1]. Over the past decade, the incidence of HCC has risen rapidly because of the expansion of HCC risk factors, including chronic viral hepatitis, excessive alcohol intake, and chemical carcinogen exposure [2]. Liver cancer has a lengthy subclinical stage, mainly due to the absence of symptoms and effective diagnostic biomarkers in the early stages. Consequently, numerous patients lose the opportunity for curative surgery, and available therapeutic options are limited or ineffective [3]. Especially, targeting classical death pathways has been unable to meet the goal. It is therefore urgent for us to develop new therapies to overcome drug resistance.

Cancers are genetically unstable and often display extensive DNA copy number aberrations (CNAs), including amplifications and deletions. Tumor suppressor gene deletion and oncogene amplification can promote aggressive cell growth and drive tumor development [4]. Furthermore, data from genome-wide CNA studies have identified additional genetic lesions that play a role in HCC progression [5]. Frequent copy number gains, suggestive of the presence of oncogenes, have been reported for chromosome region 8q in multiple cancer types [6,7]. Additionally, the common occurrence of 8q24.13–24.3 has been directly linked to worse clinical prognosis in patients with HCC because of the presence of major oncogenes, such as plasmacytoma variant translocation 1 (PVT1) [8]. Chromosome 8 open reading frame 76 (C8orf76), located in 8q24.13–24.3, was identified as a novel oncogene in gastric cancer. It was shown that C8orf76 could directly bind to oncogenic long non-coding RNA (lncRNA) dual specificity phosphatase 5 pseudogene 1 (DUSP5P1) to induce its expression and activate downstream signaling pathway mitogen-activated protein kinase (MAPK) [9]. To date, the exact molecular mechanisms of C8orf76 in most cancers including HCC are unknown and need to be investigated in depth.

In recent years, ferroptosis, which plays a potential pathogenic role in cancer, neurodegeneration, and organ dysfunction, has attracted researchers’ attention [10,11]. Ferroptosis is a novel type of regulatory cell death characterized by intracellular iron disorder and accumulation of lipid reactive oxygen species (ROS) in the cell membrane [12]. Based on accumulating findings, it was discovered that the cystine/glutamate transporter system xCT has been an increasingly important role in defending oxidative stress and ferroptosis in various types of cancers [13]. System xCT is pivotal in the exchange of cystine and glutamate, leading to the synthesis of antioxidant peptide glutathione (GSH) and phospholipid hydroperoxidase glutathione peroxidase 4 (GPX4) activation, eventually protecting cancer cells from lipid ROS attack [14]. Thus, blocking the xCT-GSH-GPX4 axis has been regarded as an encouraging means to halt tumor growth. Several compounds such as erastin and sorafenib have been appointed as direct inhibitors of xCT [15]. Additionally, repressing GPX4 activity like RSL3 is another method to provoke ferroptosis [16]. As a critical catalytic subunit of system xCT, solute carrier family 7 member 11 (SLC7A11) expression level exhibits an upward trend in multiple cancer types and is an indicator of antiporter xCT activity [17]. Therefore, more in-depth insights into the regulatory mechanisms of SLC7A11 in tumor metabolism could provide us with important clues for cancer treatment.

It is known that the tumor suppressor p53 exerts its potent antineoplastic function mainly via inducing apoptosis, senescence, and growth arrest, but several mouse models suggest that p53 stabilization sensitizes tumor cells to ferroptosis by transcriptionally downregulating SLC7A11 [18,19]. Likewise, specific transcription factor 3 (ATF3) binding to the SLC7A11 core promoter region increases cancer cell sensitivity to ferroptosis [20]. Two other well-known transcriptional factors, nuclear factor erythroid 2-related factor 2 (NRF2) and transcription factor 4 (ATF4), can bind directly to antioxidant response element (ARE) or the amino acid response element (AARE) in the promoter region of SLC7A11 and stimulate its transcription, thereby enabling cells to cope with cystine starvation [21,22]. Thus, a more thorough knowledge of the control of SLC7A11 is key to understanding cancer biology and potentiates new therapeutic targets. Here, we found that C8orf76 amplification and overexpression were found in a significant portion of HCC and correlated with poor prognosis. As a transcriptional factor, C8orf76 could activate SLC7A11 to diminish lipid peroxidative damage and induce HCC ferroptosis resistance.

## 2. Materials and Methods

### 2.1. Patients and Human Samples

We evaluated C8orf76 expression in the primary tumors and paired adjacent non-cancerous tissues of HCC patients who underwent radical surgery via immunohistochemistry (IHC), western blotting (WB), and quantitative reverse transcription PCR (RT-qPCR). Overall survival (OS) time was determined from the date of surgical resection to the end of follow-up or time of mortality. All samples were collected from patients who signed consent forms. This study the conformed to principles of the Declaration of Helsinki and was approved by the Institutional Review Board of SRRSH of Sir Run Run Shaw Hospital (SRRSH), School of Medicine, Zhejiang University, Hangzhou, China.

### 2.2. Immunohistochemistry

Tumor sections were incubated with a 1:30 dilution of C8orf76 polyclonal antibody (NBP1-82221; Novus, Littleton, CO, USA) or a 1:150 dilution of SLC7A11 polyclonal antibody (26864-1-AP; Proteintech, Wuhan, China) at 4 °C overnight for immunohistological staining. A secondary anti-rabbit IgG antibody linked to horseradish peroxidase was added and incubated at room temperature for 20 min. Finally, the reaction products were visualized with a diaminobenzidine solution, and subsequent nuclear counterstaining was performed with hematoxylin for 2 min at room temperature. Based on the degree of tissue staining, expression levels were divided into four grades (0, 1+, 2+, and 3+) and the percentages of positive cells were as follows: 0 (<5%), 1 (5–25%), 2 (25–50%), 3 (50–75%), and 4 (>75%). The final staining score was the product of both parameters and ranged from 0 to 13 points [23].

### 2.3. Lentiviral Vector Construction and Transfection

ShC8orf76-A, shC8orf76-B, and scrambled shRNA were constructed into the pLKO.1 plasmid. The human C8orf76 cDNA (C8orf76) sequence was cloned into the PCDH-FLAG lentiviral vector (Addgene, Watertown, MA, USA) for C8orf76 overexpression. Then, HEK293T cells were transiently transfected with lentiviral vectors, packaging, and envelope plasmids MD2G and PAX2 using the Lipofectamine™ 3000 transfection reagent (L3000015; Thermo Fisher, Waltham, MA, USA) to produce lentivirus supernatants. Viral supernatants were then collected, filtered through a 0.22 μm (Millipore, Burlington, MA, USA), and ultracentrifuged. After 48 h of lentivirus transfection, HCC cells were selected with puromycin treatment (1 μg/mL). Short hairpin RNA sequences for C8orf76 knockdown were as follows: shC8orf76-A, CAAGTGGTTCAGAAAGATCAA; shC8orf76-B, CAGTTCCATACAGAGATACAA.

### 2.4. Cell Lines and Regents

From the Cell Bank of the Chinese Academy of Science (Shanghai, China), SK-Hep-1 and HEK293T were purchased. Other HCC cells including HA22T, JHH7, and HCCLM3 were kindly provided by the Cang laboratory at Zhejiang University. All cell lines were cultured in DMEM medium (Gibco, Waltham, MA, USA) containing 10% fetal bovine serum (FBS, Gibco) at 37 °C in humidified air with 5% CO_2_. Erastin (S7242), RSL3 (S8155), Ferrostatin-1 (Fer-1, S7243) and Liproxstatin-1 (Lip-1, S7699) were purchased from Selleck. Sorafenib (HY-10201) was purchased from MCE.

### 2.5. Cell Viability

A total of 5000 HCC cells per well were planted into a 96-well plate and allowed to attach for 24 h. Then HCC cells were exposed to increasing doses of erastin and sorafenib for 24 h. Then, 100 μL of fresh medium containing MTS solution (0.5 mg/mL, G3580, PROMEGA, Madison, WI, USA) was added and the 490-nm absorbance was detected following incubation for 2 h at 37 °C using a spectrophotometer (Multiskan Spectrum 1500, Thermo, USA). As for the rescue assay, HCC cells were pretreated with ferroptosis inhibitors Fer-1 and Lip-1 for 1 h, then ferroptosis activators, erastin and sorafenib, were added into plates and incubated for 24 h.

### 2.6. Cell Growth Assay

1000 HCC cells were planted into 96-well plates and cultured overnight. Next, the number of cells was analyzed at indicated time (Day 0, 1, 3, 5, 7) with a 2-h incubation with the MTS reagent, which was measured at 490-nm absorbance.

### 2.7. Colony Formation Measurements

We seeded 1000 transfected HCC cells into 6-well plates. After two weeks of culture, the colonies were counted after treatment with the fixation and staining using 4% paraformaldehyde and 0.1% crystal violet.

### 2.8. Cell Cycle

5 × 10^5^ cells were collected and washed twice with PBS. Then 1 mL DNA staining solution with 10 μL permeabilization solution (PI, 70-CCS012, MultiSciences, Hangzhou, China) was added into each group for 30 min out of light at room temperature and samples were then analyzed by flow cytometry (BD LSRFortessa, New Jersey, NJ, USA).

### 2.9. PI Staining

1 × 10^5^ HCC cells were plated on a 12-well plate overnight and treated with erastin or sorafenib for 24 h. Then, the addition of Propidium iodide (PI) to per well was followed by an incubation period of 20 min at room temperature shielded from light. Thereafter, cells were viewed under an inverted fluorescence microscope (Zeiss, Oberkochen, Germany, AXIO Observer A1).

### 2.10. Tumor Xenografts

The animal experiments were strictly designed and executed according to the guide approved by the Committee of the Use of Live Animals in Teaching and Research at SRRSH. Female BALB/c nude mice (n = 5, 4 weeks old) were purchased from the Shanghai Laboratory Animal Center and fed in the Laboratory Animal Research Center of SRRSH. We injected 1 × 10^7^ control or shC8orf76 treated cells in 200 μL PBS into the axilla of nude mice to form murine subcutaneous tumors. The mice were killed two weeks after injection, and the tumors were isolated, weighed, and photographed. Finally, tumor samples were prepared as wax blocks for IHC assay.

### 2.11. Measurement of Lipid ROS and GSH

Changes in lipid peroxidation were assessed by C11- BODIPY 581/591 sensor (D3861, Invitrogen, Waltham, MA, USA) that shifted its fluorescence from red to green. HCC cells were planted in 12-well plates overnight and subjected to ferroptosis inducers (erastin, sorafenib) for 24 h. Then, 1 mL of fresh medium containing 5 μM BODIPY 581/591 C11 was added to each well. The cells were incubated for 30 min, followed by collection and analysis using a flow cytometer (BD LSRFortessa, New Jersey, NJ, USA). In addition, intracellular GSH levels were measured using a GSH colorimetric detection kit (A006-2-1, Nanjing Jiancheng Institute of Biological Engineering, Nanjing, China). Briefly, cells were collected by trypsinization, followed by washing in PBS twice. Then cells were resuspended with the addition of 300 µL PBS and broken ultrasonically to collect intracellular GSH for detection.

### 2.12. Glutamate Release Assays

Initially, 1 × 10^5^ HCC cells were seeded in 12-well plates, followed by incubation with glutamine-free medium in the presence or absence of erastin (5 μM) for 6 h. Then 50 μL supernatant media was transferred to 96-well plates and mixed with 50 μL reaction solution according to the manufacturer’s instructions (E-BC-K118-M, Elabscience, Wuhan, China). The plate was shaken for 5 s and measured at 340 nm for the first time. After 40-min incubation at 37 °C, the plate was analyzed at 340 nm again. The glutamate concentrations were calculated according to the standard curve and normalized to the total cell number based on MTS data. Results were expressed as a percentage with the negative control as 100%.

### 2.13. Fluorescence Microscope Assay

HCC cells were planted on coverslips overnight, followed by erastin or sorafenib stimulation for 24 h. Next, cultured medium was removed and incubated with C11-BODIPY 581/591 sensor (10 μM) for 30 min at 37 °C. Finally, samples were stained with DAPI and visualized under an inverted fluorescence microscope (Zeiss, AXIO Observer A1).

### 2.14. Western Blot Analysis and Antibodies

Protein expression levels were analyzed by WB according to the standard protocols. Protein extracts were run on SDS-PAGE and blotted into polyvinylidene fluoride (PVDF, Millipore) membranes. After blocking with 5% bovine serum albumin for 1 h, membranes were immunoreacted with primary antibody overnight. The primary antibodies were as follows: C8orf76 (H00084933-B01P, Abnova, Taiwan, China), SLC7A11 (#12691, Cell Signaling Technology, Danvers, MA, USA), CDK2 (ab32147, Abcam, Cambridge, UK), CDK6 (ab151247, Abcam, UK), cyclin D1 (ab226977, Abcam, UK), GAPDH (AC002, Abclonal, Wuhan, China), and FLAG (F2555, Sigma, St. Louis, MO, USA). Next, horseradish peroxidase-conjugated anti-mouse (33201ES60, Yeasen, Shanghai, China) and anti-rabbit (33101ES60, Yeasen, China) antibodies were used as secondary antibodies to probe the primary antibody, and the membranes were visualized using imaging systems.

### 2.15. Transmission Electron Microscopy

HCC cells were seeded onto 6 cm plates and treated with 7.5 μM erastin for 24 h. Then cells were fixed in 2.5% glutaraldehyde solution at 4 °C overnight. Before fixation with 1% osmic acid for 1 h, cells were washed with PBS three times. Subsequently, samples were washed with water and fixed in 2% uranium acetate for 30 min. After fixation, samples were dehydrated through an ethanol gradient. Finally, samples were embedded, ultrathin sectioned, and stained, followed by observation using a transmission electron microscopy (Tecnai G2 Spirit 120 kV, thermos FEI).

### 2.16. RNA Extraction and Quantitative Real-Time PCR

RNA extraction was performed using the RNA-Quick Purification Kit (ES Science, shanghai, China) following the manufacturer’s instructions. Reverse transcription was conducted according to Eco M-MLV RT Premix kit (AG11706, Accurate Biology, Atlanta, Georgia). Supplementary Table S1 shows the primer sequences used for RT-qPCR. Candidate gene expression was normalized to that of glyceraldehyde 3-phosphate dehydrogenase (GAPDH). RT-qPCR was performed using the SYBR Green Premix Pro Tag HS qPCR kit (AG11701, Accurate Biology) by QuantStudio 1 (applied biosystems, Thermo Fisher Scientific, Waltham, MA, USA). Samples were run and analyzed in triplicate.

### 2.17. Immunofluorescent Staining

After seeding on coverslips overnight, HCC cells were fixed in 4% paraformaldehyde before permeabilization with 0.5% Triton X-100 and blocking with 5% BSA. The slides were treated with primary anti-FLAG antibody overnight at 4 °C, followed by incubation with fluorescence-conjugated secondary antibody at room temperature for 1 h and nuclei staining with DAPI. Fluorescent images were collected using a laser scanning confocal microscope (TCS SP8, Leica, Wetzlar, Germany).

### 2.18. Chromatin Immunoprecipitation (ChIP)

CHIP assay was carried out using a ChIP assay kit (P2078, Beyotime, Shanghai, China). Briefly, 1% formaldehyde was added to the medium to crosslink chromatin and protein. Then DNA-protein cross-links were sonicated into ~500 bp fragments and immunoprecipitated with FLAG antibody overnight followed by pull-down using Protein A-Sepharose beads. Finally, immunoprecipitated DNA fragments were purified using the ChIP Assay Kit (D0033, Beyotime) and analyzed using RT-qPCR as described above. Supplementary Table S1 lists the specific primers for the SLC7A11 promoter.

### 2.19. Luciferase Assay

HCC cells were co-transfected with PGL3-SLC7A11 promoter constructs along with a Renilla luciferase-expressing plasmid. 48 h post-transfection culture, the firefly luciferase intensity was measured using a Dual-Luciferase Reporter Gene Assay Kit (11402ES60, Yeasen, China) and normalized to the Renilla intensity following the manufacturer’s instructions. The sequence for the PGL3-SLC7A11 promoter construct was listed in Supplementary Table S2.

### 2.20. Statistical Analysis

Statistical significance between the two groups was calculated with the Student’s t-test. Variances among multiple groups were analyzed by one-way or two-way ANOVA. A χ^2^ test was used to compare the patients’ clinical features and C8orf76 expression. The survival curves’ significance for Kaplan-Meier plots was assessed using the log-rank test for trend. Pearson’s correlation analysis was performed to calculate the correlation between the two groups. *p* < 0.05 was considered statistically significant.

## 3. Results

### 3.1. C8orf76 Expression Was Enhanced in HCC Patients and Related to HCC Progression and Poor Clinical Outcomes

**Figure 1:**
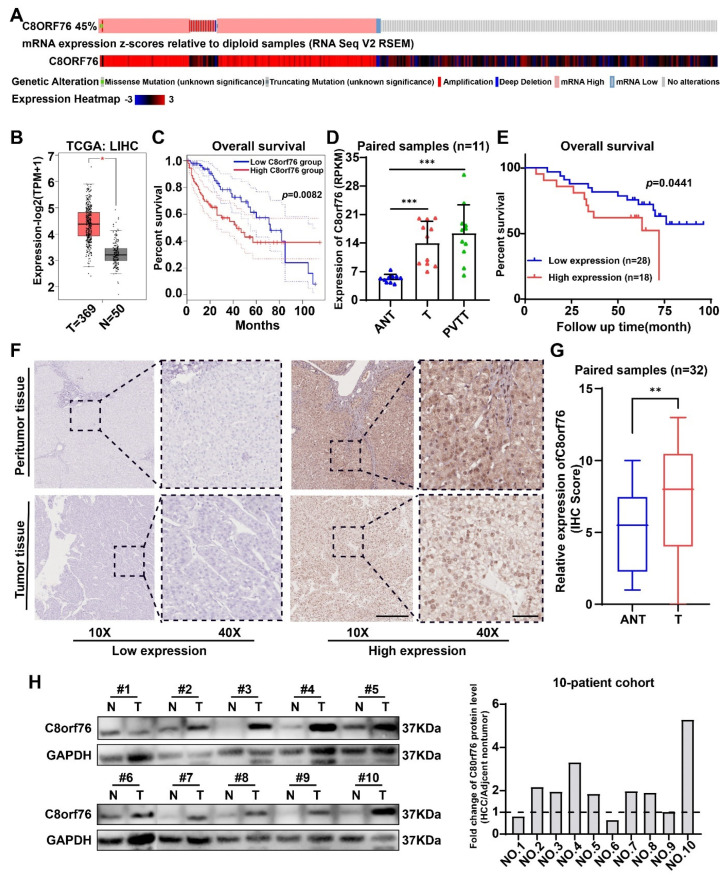
C8orf76 expression is upregulated in HCC tumors and correlates with HCC progression and prognosis. (**A**) The association of C8orf76 DNA copy−number amplification and its mRNA expression in HCC patients of TCGA. (**B**) The mRNA levels of C8orf76 were analyzed in HCC tissues of the TCGA dataset compared to the normal controls. (**C**) Kaplan−Meier analysis of OS for all HCC patients with low or high C8orf76 expression level in TCGA dataset. (**D**) C8orf76 was analyzed in our RNA-seq dataset including 11 paired HCC samples. (**E**) Kaplan−Meier analysis of HCC OS based on C8orf76 protein expression level in HCC tissues (n = 46). (**F**) Representative images of C8orf76 expression in HCC and adjacent tissues. (**G**) The evaluation of C8orf76 expression by performing an IHC staining analysis of 32 paired HCC tissue samples. Scale bars represent 250 μm (left) and 50 μm (right). (**H**) Protein expression of C8orf76 in HCC and matched noncancerous tissues through western blot analysis. Data were presented as mean ±SD. * *p* < 0.05; ** *p* < 0.01; *** *p* < 0.001; ns, not significant. T, tumor, ANT, adjacent nontumor, PVTT, portal vein tumor thrombosis.

Based on the data from The Cancer Genome Atlas (TCGA) datasets, high C8orf76 mRNA expression was found in liver cancer patients compared with corresponding paracancerous tissues (Figure 1B). Meanwhile, there was a positive correlation with copy number amplification in patients with C8orf76 upregulation (Figure 1A). This suggests that C8orf76 copy number gain commonly resulted in its overexpression. Notably, patients with increased C8orf76 had worse OS in the TCGA dataset (Figure 1C). Moreover, we also found that C8orf76 was highly expressed in tumor and portal vein tumor thrombosis (PVTT) tissues compared to adjacent benign tissues according to our previous RNA-seq analysis [24] (Figure 1D).

Next, IHC was performed to analyze C8orf76 protein expression and location in human HCC tissues. As Figure 1F showed, C8orf76 was detected in both tumor and adjacent normal tissues. However, expression levels of C8orf76 were found to be relatively high in liver cancer, whereas it was minimally expressed in adjacent normal tissues (Figure 1G). Kaplan–Meier survival analysis demonstrated that higher C8orf76 expression was significantly predictive of worse OS for HCC patients (Figure 1E). The significance of the C8orf76 expression and clinicopathological parameter correlations were determined by a χ^2^ test (Table 1). The results indicated that C8orf76 expression was positively associated with alpha-fetoprotein (AFP) level (*p* = 0.036) and vascular invasion (*p* = 0.025). Furthermore, the WB assay also confirmed that C8orf76 was highly expressed in HCC compared with noncancerous tissues (Figure 1H). Cumulatively, these findings suggested that C8orf76 had significantly higher expression in HCC tissues and was associated with an advanced disease stage and poor prognosis.

### 3.2. C8orf76 Knockdown Inhibited HCC Cell Growth and Cell-Cycle Progression

**Figure 2:**
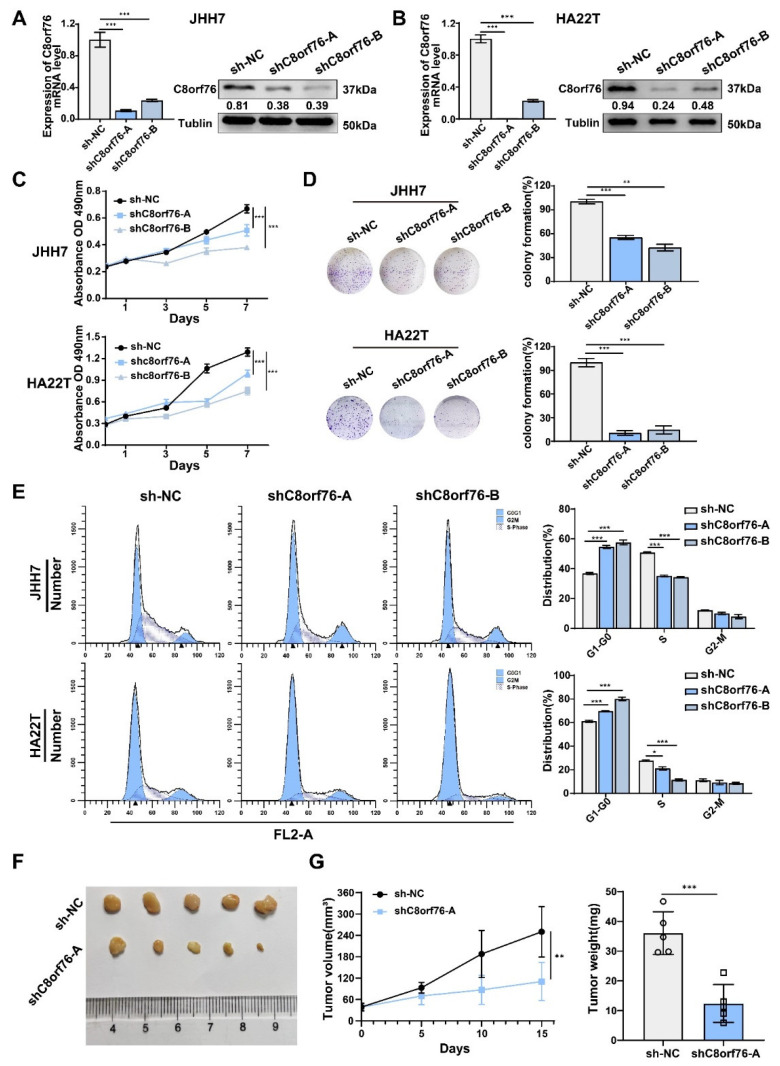
C8orf76 knockdown inhibits liver cancer cell growth, colony formation and cell cycle. (**A**,**B**) Relative C8orf76 mRNA and protein level in HCC cell lines with or without shC8orf76 transfection (n = 3). (**C**,**D**) C8orf76 knockdown significantly inhibited cell proliferation and impaired colony formation (n = 3). (**E**) Depletion of C8orf76 caused G1–S arrest of HCC cells (n = 3). (**F**) Representative photographs of tumor growth in nude mice subcutaneously inoculated with shC8orf76- or shNC-transfected JHH7 cells were shown by growth curve of tumor volume, and tumor weight (**G**) at the end of the experiment (n = 5). Data was presented as mean +SD. * *p* < 0.05; ** *p* < 0.01; *** *p* < 0.001; ns, no significant.

Since C8orf76 was regarded as an oncogene in clinical samples and public cancer data, we first evaluated the effects of C8orf76 on HCC tumor growth in vitro. C8orf76 was knocked down by lentiviral transfection in HCC cells, and downregulation efficiency was verified by RT-qPCR and WB (Figure 2A,B). MTS assays indicated that C8orf76 repression in HCC cell lines led to a marked reduction in cell proliferation (Figure 2C). Consistently, the clone-forming ability of HCC cells was inhibited after C8orf76 knockdown (Figure 2D). Meanwhile, an obvious association between C8orf76 status and Ki67 and proliferating cell nuclear antigen (PCNA) expression was seen in the TCGA dataset (Supplementary Figure S1). The results implied that the C8orf76 gene was closely associated with HCC malignant features. Next, flow cytometry was conducted to analyze the effect of C8orf76 on the cell cycle. C8orf76-depleted HCC cells were arrested in the G1/S phase (Figure 2E). Key proteins regulating cell cycle progression from G1 to S phase, including cyclin D, CDK6, and CDK2, were examined upon C8orf76 depletion. As Figure S2A,B showed, the protein levels of these target genes (cyclin D, CDK6, CDK2) were significantly decreased in C8orf76-knockdown cells.

Additionally, a subcutaneous xenograft tumor model in nude mice was established by transplanting HCC cells treated with sh-NC and shC8orf76 in the axilla of nude mice. Tumors in the stable C8orf76 depletion group appeared smaller in size and weighed less than tumors resulting from control cells (Figure 2F,G). Altogether, these findings suggested that C8orf76 positively regulated HCC proliferation.

### 3.3. C8orf76 Knockdown Rendered HCC Cells Susceptible for Ferroptosis

**Figure 3:**
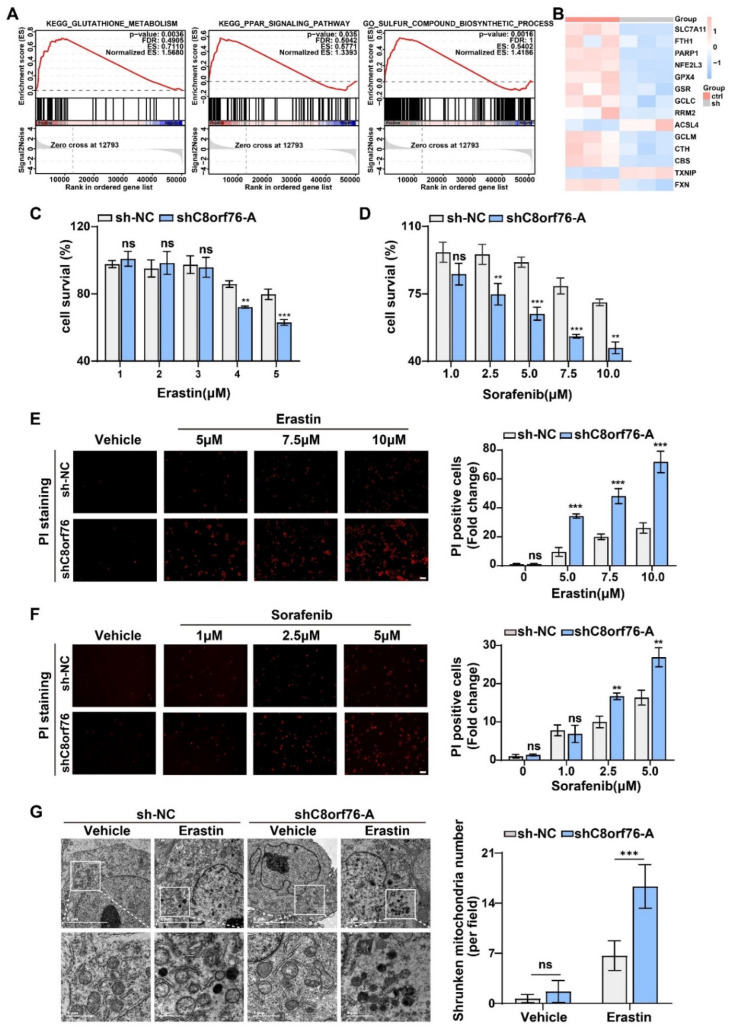
C8orf76 knockdown renders HCC cells susceptible for erastin and sorafenib. (**A**) Gene set enrichment analysis related to C8orf76 functions. (**B**) The heatmap of altered ferroptosis-associated genes. (**C**,**D**) JHH7 cells were exposed to different doses of erastin or sorafenib for 24 h and detected by MTS reagent. (**E**,**F**) The degree of the cell-membrane injury induced by erastin or sorafenib for 24 h was stained by propidium iodide and viewed under inverted fluorescent microscope. Scale bars represent 100 μm. (**G**) Transmission electron microscopy images of mitochondria in C8orf76-depleted JHH7 cell line. Data were presented as mean +SD. ** *p* < 0.01; *** *p* < 0.001; ns, not significant.

We performed RNA-seq analysis between the control group and C8orf76 knockdown HCC cells to elucidate the molecular mechanism by which C8orf76 functions. Subsequently, a heatmap of differentially expressed genes (DEGs) displayed the overall changes (Figure S3A). Kyoto Encyclopedia of Genes and Genomes (KEGG) enrichment revealed that critical tumor-associated pathways were significant difference while C8orf76 knockdown (Figure S3B). However, several DEG enrichment analysis limitations should be noted because some essential genes with minor changes may have gone unnoticed. Thus, gene set enrichment analysis (GSEA) was conducted to confirm the enrichment patterns of genes related to C8orf76 functions. Surprisingly, the results demonstrated that ferroptosis-related pathways were remarkably correlated with C8orf76 alteration, including GSH metabolism, the peroxisome proliferator-activated receptor (PPAR) signaling pathway, and the sulfur compound biosynthetic process [25,26,27] (Figure 3A). In addition, there was an obvious expression difference of critical ferroptosis-associated genes between the control and knockdown groups (Figure 3B). Therefore, we inferred that C8orf76-mediated ferroptosis might exert an important role in progressing HCC to a greater degree.

Ferroptosis is a novel form of programmed cell death that is closely linked to iron metabolism. It is implicated in tumor pathogenesis including HCC and can be induced by erastin or sorafenib through targeting cystine/glutamate antiporter [28,29]. Proliferation assays indicated that depletion of C8orf76 sensitized HCC cells to erastin-induced death in a dose-dependent manner (Figure 3C). Similarly, we observed that C8orf76 knockdown also coordinated with sorafenib to promote cell death (Figure 3D). As the rupture of membrane permeability is a characteristic of ferroptosis, we performed PI staining to reflect the degree of the cell-membrane injury and cell death. As depicted in Figure 3E,F, treatment of erastin and sorafenib remarkably increased the pool of dead cells in the knockdown group. We further performed a transmission electron microscopy assay to reveal the mitochondrial morphology while C8orf76 knockdown. Although there was no obvious difference in morphological features in JHH7 cells treated with DMSO, C8orf76-depleted cells displayed shrunken mitochondria, increased membrane density, and cristae thickening when treated with erastin (Figure 3G).

**Figure 4:**
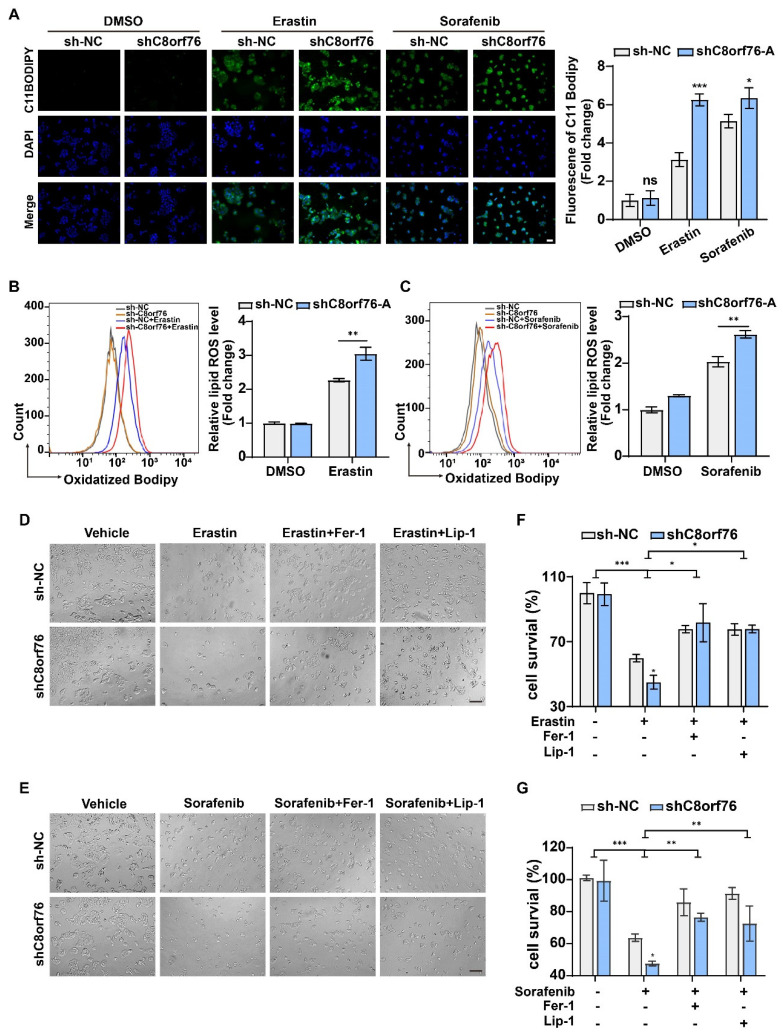
C8orf76 knockdown renders cells susceptible for ferroptosis and lipid ROS accumulation. (**A**) Representative images of C11−BODIPY staining in JHH7 cells after erastin and sorafenib treatment were photographed by an inverted fluorescent microscope. Scale bars represent 50 μm. (**B**,**C**) Flow cytometry was performed to assess the lipid ROS accumulation of JHH7 cells treated with erastin or sorafenib for 24 h using C11−BODIPY. (**D**,**E**) Representative images of HCC cells treated with erastin (7.5 μM) or sorafenib (5 μM) with or without Fer−1 (1 μM) or Lip−1 (0.4 μM) for 24 h. Scale bars represent 200 μm. (**F**,**G**) The rescue effect of ferroptosis inhibitors on erastin or sorafenib treatment was explored through an MTS assay. Data were presented as mean ±SD. * *p* < 0.05; ** *p* < 0.01; *** *p* < 0.001; ns, not significant. Fer-1, ferrostatin-1; Lip-1, liproxstatin-1.

The fluorescent probe, C11-BODIPY 581/591, was used as a lipid peroxidation-sensitive dye by a fluorescent microscope and flow cytometer. Following the induction of erastin and sorafenib after 24 h, the green fluorescent intensity was obviously enhanced in the C8orf76^KD^ group under the inverted fluorescent microscope (Figure 4A). Using flow cytometric analysis, we also confirmed that lipid peroxidation was indeed increased in the C8orf76-depleted group that was directly exposed to erastin and sorafenib (Figure 4B,C). However, values were not significantly different between the control and knockdown group without ferroptosis inducers. In order to investigate whether C8orf76 depression could augment the process of ferroptosis specifically, we monitored the rescue effect induced by erastin or sorafenib with and without various ferroptosis inhibitors. JHH7 cells were exposed to erastin (7.5 μM) for 24 h. It was found that erastin treatment could significantly exert a growth inhibitory effect in C8orf76^KD^ cells. Notably, erastin-induced cell growth inhibitory could be restored by ferroptosis inhibitors Fer-1 (1 μM) and Lip-1 (0.4 μM) (Figure 4D,F). Comparable results were also obtained when the ferroptosis inducer, sorafenib, was combined with ferroptosis inhibitors (Figure 4E,G). We further explored the effects of GPX4 inhibitor (RSL3) on ferroptosis. Intriguingly, C8orf76 knockdown remarkably promoted RSL3-induced cell death, which could also be suppressed by Fer-1 (1 μM) and Lip-1 (0.4 μM) (Figure S4A,B). Collectively, these data indicate that C8orf76 deficiency could accelerate cell ferroptosis provoked by ferroptosis inducers.

### 3.4. C8orf76 Overexpression Mediated Tumor Ferroptosis Resistance

**Figure 5:**
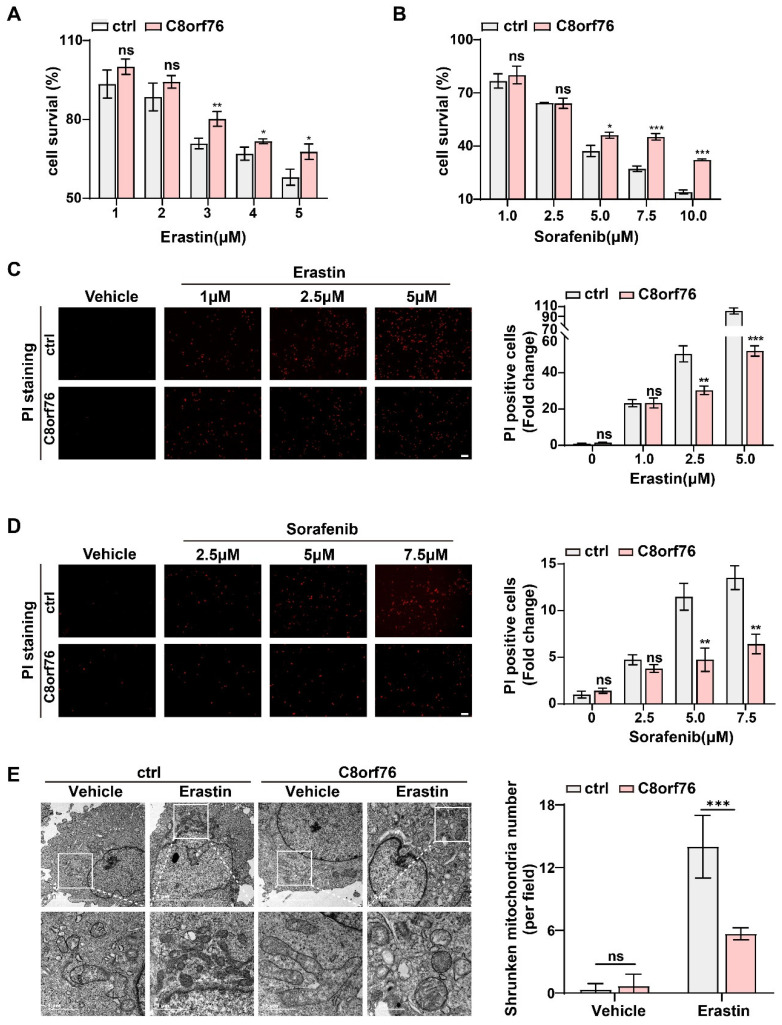
C8orf76 overexpression mediated tumor resistance towards erastin and sorafenib. (**A**,**B**) Erastin or sorafenib treatment resulted in a dose-dependent inhibition of cell viability. Cell survival was measured by MTS. (**C**,**D**) PI staining showed the number of dead cells following the treatment of erastin at the concentration of 1 μM, 2.5 μM, and 5 μM or sorafenib at the concentration of 2.5 μM, 5 μM, and 7.5 μM for 24 h. Scale bars represent 100 μm. (**E**) C8orf76-overexpressed SK-Hep-1 cells were treated with erastin and analyzed by transmission electron microscopy. Data were presented as mean +SD. * *p* < 0.05; ** *p* < 0.01; *** *p* < 0.001; ns, not significant.

HCC cells transfected with lentivirus achieved stable C8orf76 overexpression. RT-qPCR and WB also verified this efficiency (Figure S5A,B). Under basal conditions, no significant differences were present in cell growth between the C8orf76 overexpression and control groups in SK-Hep-1 and HCCLM3 cells (Figure S5C–F). Then, we attempted to explore the extent to which ferroptosis stimulation could influence tumor cells after C8orf76 upregulation. As shown in Figure 5A, different doses of erastin significantly inhibited cell proliferation, which can be blunted by ectopic C8orf76 expression. In contrast to the basal condition, C8orf76 was also found to predict sorafenib treatment efficacy (Figure 5B). Compared to the control group, the cell-membrane damage upon erastin was reduced by PI staining in the C8orf76 overexpression group (Figure 5C). Likewise, the injury degree of the cell membrane upon exposure to sorafenib was also restored by C8orf76 overexpression (Figure 5D). Moreover, transmission electron microscopy revealed that ectopic expression of C8orf76 could keep the integrity of mitochondrial morphology even in the treatment of erastin. However, typical ferroptosis ultrastructural changes including shrunken mitochondria with elevated membrane density were shown in the control group induced by erastin (Figure 5E).

**Figure 6:**
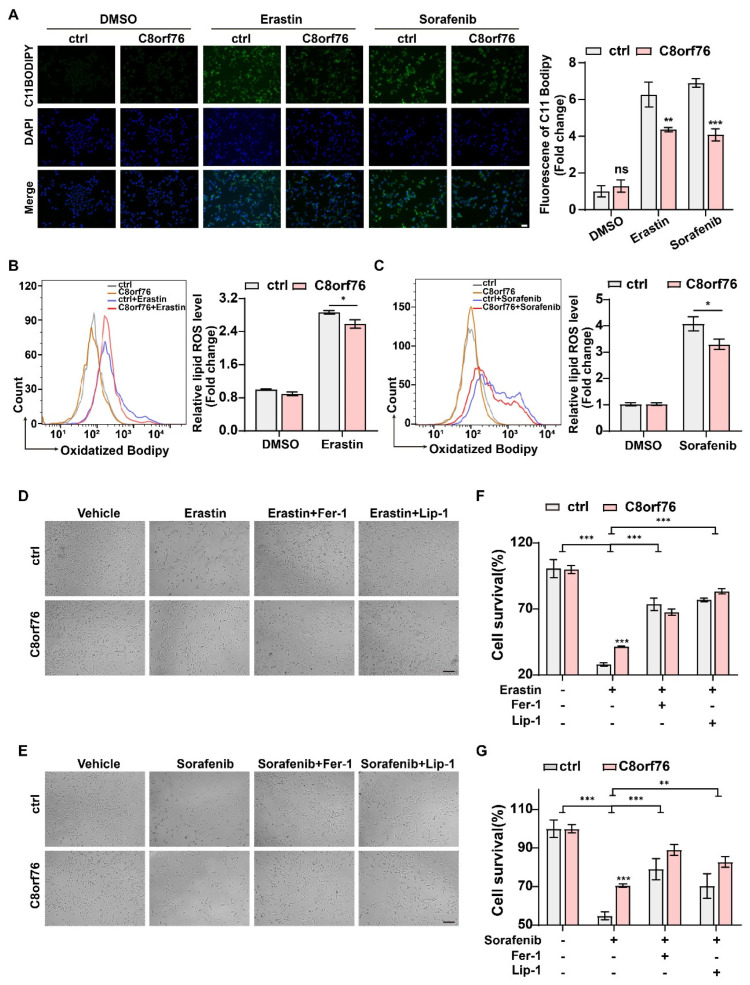
Erastin and sorafenib-induced accumulation of lipid ROS and ferroptois in C8orf76OE cells. Lipid ROS production followed by erastin and sorafenib was viewed by an inverted fluorescent microscope (**A**) and detected by flow cytometry (**B**,**C**). Scale bars represent 50 μm. (**D**,**E**) HCC cells were visualized at the treatment of 7.5 μM erastin or 7.5 μM sorafenib with or without ferroptosis inhibitors (Fer−1, 1 μM; Lip−1, 0.4 μM). Scale bars represent 200 μm. (**F**,**G**) MTS assay was performed to monitor the cell viability induced by erastin (7.5 μM) or sorafenib (7.5 μM) combined with or without ferroptosis inhibitors (Fer−1, 1 μM; Lip−1, 0.4 μM). Data were presented as mean +SD. * *p* < 0.05; ** *p* < 0.01; *** *p* < 0.001; ns, not significant. Fer−1, ferrostatin−1; Lip−1, liproxstatin−1.

Intriguingly, fluorescent intensity in C8orf76^OE^ cells stained by C11-BODIPY 581/591 was reduced while treatment with erastin or sorafenib (Figure 6A). Consistent with previous data, flow cytometric analysis indicated that erastin and sorafenib-elicited accumulation of lipid peroxidation exhibited a downward trend after C8orf76 overexpression in comparison with the control group (Figure 6B,C). These results demonstrated that C8orf76 overexpression could significantly diminish lipid peroxide induction in HCC cells followed by ferroptosis inducers. Correspondingly, as Figure 6D,E showed, while C8orf76 upregulation decreased erastin-induced cell viability inhibition, co-treatment with Fer-1 (1 μM) or Lip-1 (0.4 μM) was of significance for the prevention of erastin-induced cell death in control and C8orf76^OE^ group. In line with erastin effect, similar rescue results were also observed when sorafenib was applied (Figure 6F,G). Furthermore, a significant variation of cell viability was seen between C8orf76^OE^ cells and controls upon treatment with RSL3 (Figure S6A). However, combination treatment with RSL3 and ferroptosis inhibitors (Fer-1 and Lip-1) markedly blocked RSL3-induced cell death (Figure S6B). Overall, our findings suggested that C8orf76 overexpression could reduce ferroptosis triggered by ferroptosis activators through improving membrane lipid peroxidation.

### 3.5. C8orf76 Regulated xCT Activity and Glutamate Secretion

**Figure 7:**
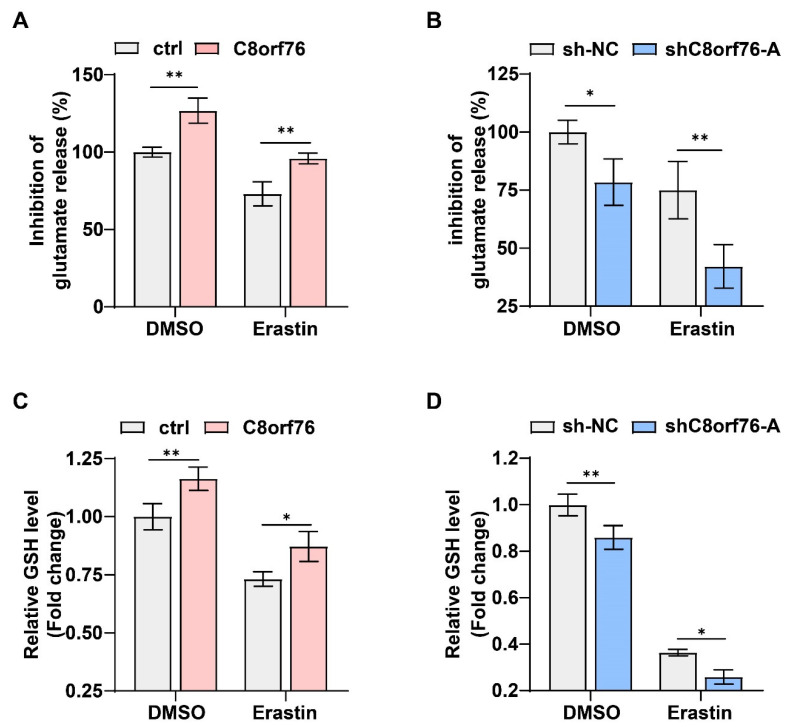
C8orf76 regulates xCT activity and glutamate secretion. (**A**,**B**) While modulating C8orf76 expression, the amount of glutamate released into culture medium was detected after treatment with or without erastin (5 μM) in HCC cells for 6 h. (**C**,**D**) Intracellular GSH levels were measured in HCC cells with C8orf76 alteration in the presence or absence of erastin (5 μM) for 12 h. Data was presented as mean +SD. * *p* < 0.05; ** *p* < 0.01; ns, no significant.

As mentioned above, the GSEA of the curated gene set revealed a dramatic impairment in GSH metabolism pathways. System xCT-cystine/glutamate antiporter, an important transporter located on cell membranes, is responsible for the uptake of extracellular cystine and the release of intracellular glutamate. Activating antiporter can lead to an increase of intracellular cysteine, thus elevating the biosynthesis of GSH and impeding ferroptosis [30]. To validate the underlying mechanism via which C8orf76 mediated ferroptosis, we examined system xCT activity through measuring the extracellular glutamate secreted by cultured cells. As expected, a higher concentration of glutamate released into the extracellular space was shown in the C8orf76^OE^ group even in the absence of erastin (Figure 7A). In contrast, suppression of C8orf76 could obviously reduce glutamate secretion under the same condition (Figure 7B). In line with previously described experiments, C8orf76 overexpression led to an increase in GSH biosynthesis while deletion of C8orf76 attenuated cellular GSH levels regardless of erastin presence (Figure 7C,D). Therefore, we hypothesized that C8orf76 might inhibit ferroptosis through activating system xCT.

### 3.6. C8orf76 Bound to the Promoter of SLC7A11 and Promoted Its Transcription

**Figure 8:**
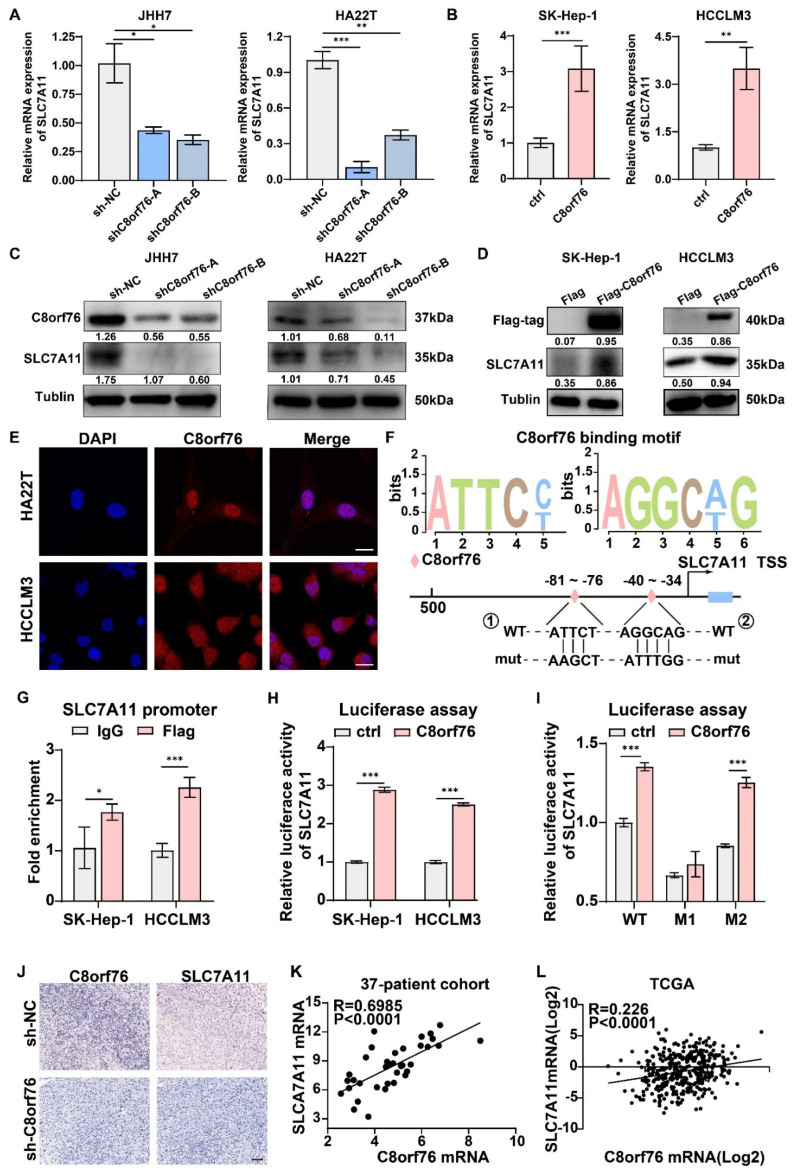
C8orf76 activates SLC7A11 at the transcriptional level through directly binding to the promoter of SLC7A11. (**A**,**B**) SLC7A11 was analyzed using qPCR in HCC cells stably knocking down or overexpression of C8orf76 (n = 3). (**C**,**D**) The protein expression of SLC7A11 was analyzed by Western blot after C8orf76 knockdown or overexpression in HCC cells. (**E**) The localization of C8orf76 was viewed by laser scanning confocal microscope in both HA22T and HCCLM3 cells. Scale bars represent 20 μm. (**F**) Two potential C8orf76 DNA−binding motifs in the promoter region of SLC7A11. (**G**) CHIP-qPCR analysis was performed to determine the relation between C8orf76 and the promoter of SLC7A11. (**H**) The relative activities of the SLC7A11 promoter after transfection of C8orf76 and vector. (**I**) The relative activities of the SLC7A11 promoter and the mutant promoter after transfection of C8orf76 and vector. (**J**) C8orf76 and SLC7A11 protein expression in subcutaneous xenografts detected by immunohistochemistry. Scale bars represent 50 μm. (**K**,**L**) C8orf76 mRNA level is positively correlated with SLC7A11 mRNA levels in 37 patients and the TCGA dataset. Data were presented as mean +SD. * *p* < 0.05; ** *p* < 0.01; *** *p* < 0.001; ns, not significant. WT, wild type; M1, mutated C8orf76 recognition site 1; M2, mutated C8orf76 recognition site 2.

The SLC7A11 gene encodes a cystine/glutamate xCT transporter, which is of paramount importance in maintaining intracellular GSH levels and alleviating cell peroxidation [13]. Thus, we sought to test whether C8orf76 could drive SLC7A11 expression. Transcriptional levels of SLC7A11 were validated by RT-qPCR in HCC cells with stable C8orf76 knockdown or upregulation. It was shown that SLC7A11 expression changed consistently with C8orf76 alteration (Figure 8A,B). We also assessed the relative expression of SLC7A11 protein by WB to verify the results indicated in C8orf76-deficient or C8orf76-overexpressing HCC cells. Results indicated a positive correlation between SLC7A11 expression and C8orf76 (Figure 8C,D).

Predict Protein website potentiated that C8orf76 belonged to the zinc-finger transcription factor family and was mainly distributed in the nucleus (Supplementary Figure S7). Therefore, we first verified the nuclear accumulation of the C8orf76 protein by immunofluorescence in both HA22T and HCCLM3 cells transfected with C8orf76 (Figure 8E). An earlier study identified five typical nuclear factor of activated T-cells (NFAT) core motifs as the potential binding sites of C8orf76, including CACACACACACACACACACACACACACAC, ACCCCCAG, AGGCT/AG, ATTCC/T, and CCAGCCAA [9]. After screening the SLC7A11 gene’s promoter region, we found that two potential C8orf76 DNA-binding motifs existed in the SLC7A11 promoter region, indicating that SLC7A11 may be an important C8orf76 target (Figure 8F). We then designed specific primers and performed a ChIP-qPCR analysis to investigate if the SLC7A11 promoter was affected by C8orf76 according to the C8orf76 binding motif. As Figure 8G showed, C8orf76 was noticeably enriched in the SLC7A11 promoter binding region. Next, we used a dual-luciferase reporter system to validate the C8orf76 modulatory effect on SLC7A11. In line with the RT-qPCR and ChIP-qPCR results, luciferase assays showed that the luciferase activities of the SLC7A11 promoter construct were markedly enhanced compared to the control vector, and mutation of binding site 1 rather than site 2, abrogated the SLC7A11 promoter reporter activity induced by C8orf76 overexpression (Figure 8H,I). Moreover, in vivo findings revealed that reduced SLC7A11 expression was also detected in shC8orf76 xenograft tumors when compared with the control group (Figure 8J). Furthermore, a notable positive correlation was observed between C8orf76 and SLC7A11 expression in our own HCC patient tissues (Figure 8K). Moreover, according to the TCGA HCC dataset, SLC7A11 was also positively associated with C8orf76 expression (Figure 8L). Collectively, C8orf76 could bind to its DNA-binding site in the promoter region of SLC7A11 and positively mediate SLC7A11 expression at the transcriptional level.

## 4. Discussion

The present study identified the potential role and molecular basis of C8orf76 in HCC cell ferroptosis. By analyzing the publicly available TCGA database and our HCC cohorts, a higher mRNA level of C8orf76 was shown in HCC tissues compared with the ANT, and patients with increased C8orf76 levels displayed worse OS. Meanwhile, C8orf76 overexpression was positively associated with copy number amplification in HCC samples based on the TCGA dataset. Importantly, we confirmed the effect of C8orf76 on liver cancer ferroptosis by altering C8orf76 expression. Mechanistically, C8orf76 knockdown repressed SLC7A11 transcription and decreased SLC7A11-dependant cystine import, which eventually retarded GSH synthesis and triggered HCC cell ferroptosis.

Within many forms of cell death-regulated pathways, ferroptosis is an iron-dependent form of nonapoptotic cell death process occurred by unrestrained lipid peroxidation [12]. Recently, there is sufficient evidence to establish ferroptosis as a tumor suppression mechanism [31,32]. Ferroptosis promotion triggered by erastin or sorafenib is mainly due to interference with system xCT membrane transporter function [12,33]. As reported previously, SLC7A11, which encodes a vital element of the antiporter system xCT, is a critical protein that controls cystine import in exchange for intracellular glutamate [13]. SLC7A11 is able to take up extracellular cystine and subsequently reduce it to cysteine rapidly, which acts as a precursor for GSH production. GPX4 uses GSH as a hydrogen donor to produce lipid hydroperoxides. Therefore, SLC7A11 inhibition or cystine removal in the culture medium could significantly provoke potent ferroptosis in many cancer cells [34]. The signal transducers and activators of transcription (STAT) family members consist of distinct genes including STAT1, STAT2, STAT3, STAT4, and STAT5 [35]. It has been reported that STAT3 and STAT5A are bound to the SLC7A11 promoter and negatively regulate basal SLC7A11 expression. Pharmacologically suppressing STAT3/5 activation could induce an adaptive, compensatory mechanism to protect breast cancer cells from ROS by activating SLC7A11 transcription and the action of system xCT [36]. AT-rich interaction domain 1A (ARID1A) encodes a protein that forms a subunit of the SWI/SNF chromatin-remodeling complex and its mutation has frequently occurred in multiple cancer types. ARID1A-deficient cancer cells appear to present a relatively low profile of SLC7A11 because the SWI/SNF complex recruitment to the promoter of SLC7A11 is dramatically reduced, which results in the impediment of GSH synthesis [37]. In conclusion, the biological function and regulation of SLC7A11 will attract significant attention in cancer research, and targeting the SLC7A11 pathway may provide a novel and effective therapeutic approach for anticancer therapy. In this study, we identified C8orf76 as a novel SLC7A11 gene regulator at the transcriptional level, which provided new insights into the mechanism of ferroptosis in cancer.

It is well known that chromosome arm 8q gains commonly occur in liver cancer [5]. C8orf76, a protein expressed in the nucleus, is located in 8q24.13-24.3, an HCC segment [8]. A previous study confirmed that C8orf76 acted as a tumor-promoting role in gastric cancer that transactivated lncRNA DUSP5P1 by directly binding to the DUSP5P1 promoter and activating the MAPK/ERK signaling pathway [9]. Here, we investigated the association between the C8orf76 expression and HCC features. In addition to its effect on cell cycle and proliferation, we surprisingly found that C8orf76 deficiency could increase intracellular lipid ROS and provoked HCC ferroptosis in the presence of erastin or sorafenib. Typical mitochondrial morphology features of ferroptosis were shown in the C8orf76-depleted group following erastin treatment. In contrast, high C8orf76 abundance prevented HCC cell death elicited by xCT inhibitors and blocked erastin-induced mitochondria morphological damage. Meanwhile, ferroptosis inhibitors specifically restored cell survival provoked by ferroptosis activators. Apart from xCT inhibitors, C8orf76 also regulated the HCC response level to GPX4 inhibitors such as RSL3. Since RNA-seq analysis indicated that overall changed genes after C8orf76 deficiency were significantly enriched in the GSH metabolism pathway, we confirmed that C8orf76 could increase cystine/glutamate exchange ratio through activating system xCT activity directly, eventually contributing to increased GSH synthesis and cell membrane protection. Furthermore, as a pivotal component of cystine/glutamate antiporter system xCT, mRNA and protein levels of SLC7A11 were changed consistently after C8orf76 interference or overexpression. Mechanistically, C8orf76, as a transcription factor, can bind to the SLC7A11 promoter region to transactivate its expression and result in system xCT activation. Taken together, our results demonstrated that C8orf76 could positively modulate SLC7A11 expression at the transcriptional level and might be a novel driver during HCC cell growth.

## 5. Conclusions

In summary, we demonstrated that C8orf76 was highly expressed and acted as an oncogene in HCC. We provided the first evidence showing that C8orf76 was involved in ferroptosis regulation via transcriptional SLC7A11 activation to investigate the concrete mechanism of C8orf76 during liver cancer progression. The crosstalk between C8orf76-mediated gene transcription and GSH metabolism may have broad implications for modulating HCC ferroptosis, suggesting that targeting the C8orf76/SLC7A11 pathway may improve anticancer therapy via ferroptosis induction.

## Figures and Tables

**Table 1:** Correlation of C8orf76 expression and clinical features in HCC patients.

Variable	C8orf76			
	Sum	Low Expression	High Expression	*p*-Value ^a^
Age				0.607
<50	15	8	7	
≥50	31	19	12	
Gender				0.355
Male	39	24	15	
Female	7	3	4	
HBV				0.685
Negative	21	13	8	
Positive	25	14	11	
AFP (ng/mL)				**0.036**
<400	32	22	10	
≥400	14	5	9	
Cirrhosis				0.655
No	26	16	10	
Yes	20	11	9	
Tumor size (cm)				0.362
T < 3	13	9	4	
T ≥ 3	33	18	15	
Vascular invasion				**0.025**
No	40	26	14	
Yes	6	1	5	
LNM				0.810
No	38	22	16	
Yes	8	5	3	
TNM stage				0.655
I–II	20	11	9	
III–IV	26	16	10	

^a^ Chi-square test, AFP alpha-fetoprotein, HBV Hepatitis B Virus, LNM lymph node metastasis. Bold values indicate statistical significance, *p* < 0.05.

## Data Availability

The data presented in this study are available in the article and supplementary files.

## Supplementary Materials

The following supporting information can be downloaded at: https://www.mdpi.com/article/10.3390/cancers14143410/s1, Figure S1: The correlation between C8orf76 level and expression of Ki67 and PCNA in TCGA HCC dataset. Figure S2: The levels of key regulators of G1/S transition were examined upon C8orf76 depleted. Figure S3: (A) The overall gene expression changes in HA22T cells with or without shC8orf76 infection, and upregulated genes presented in red while downregulated genes in blue (n = 3). (B) The KEEG pathway enrichment analysis upon those changed genes with |log2FC|>1. Figure S4: C8orf76 knockdown sensitizes HCC cells to GPX4 inhibitor-induced ferroptosis. Figure S5: Analysis of cell growth in HCC upon C8orf76 overexpression. Figure S6: C8orf76 overexpression mediated ferroptosis resistance towards GPX4 inhibitor. Figure S7: Online prediction of C8orf76 distribution in cells (PredictProtein). Table S1: DNA sequences of primers used in this study. Table S2: The sequence for the PGL3-SLC7A11 promoter construct.
