# Supplementary material for: C8orf76 Modulates Ferroptosis in Liver Cancer via Transcriptionally Up-Regulating SLC7A11
Source: Cancers (Basel). 2022 Jul 13;14(14):3410. doi: 10.3390/cancers14143410 (PMC9316296; doi:10.3390/cancers14143410)
Supplement: Supplementary file 1 [file cancers-14-03410-s001.zip › cancers-1799964-supplementary.pdf]

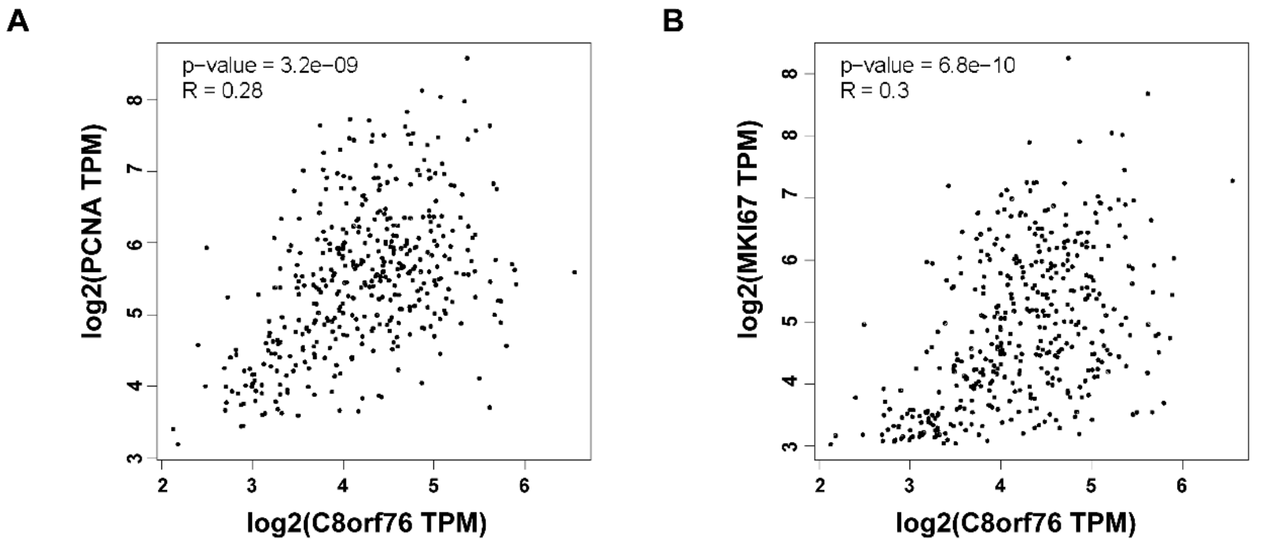

Figure S1. (**A**,**B**) The correlation between C8orf76 level and expression of Ki67 and PCNA in TCGA HCC dataset.

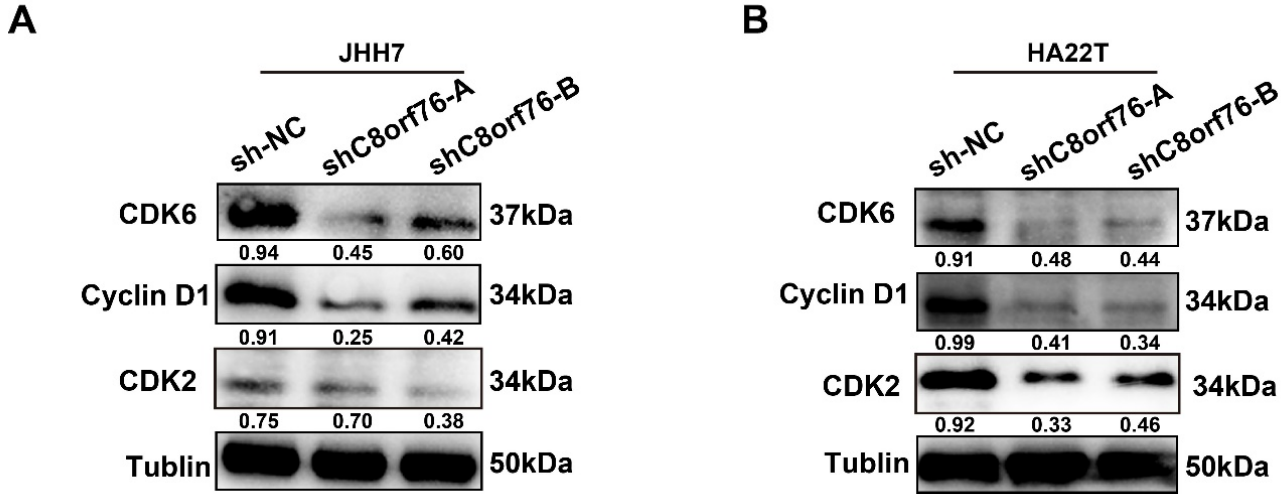

Figure S2. (**A**,**B**) The levels of key regulators of G1/S transition were examined upon C8orf76 depleted.

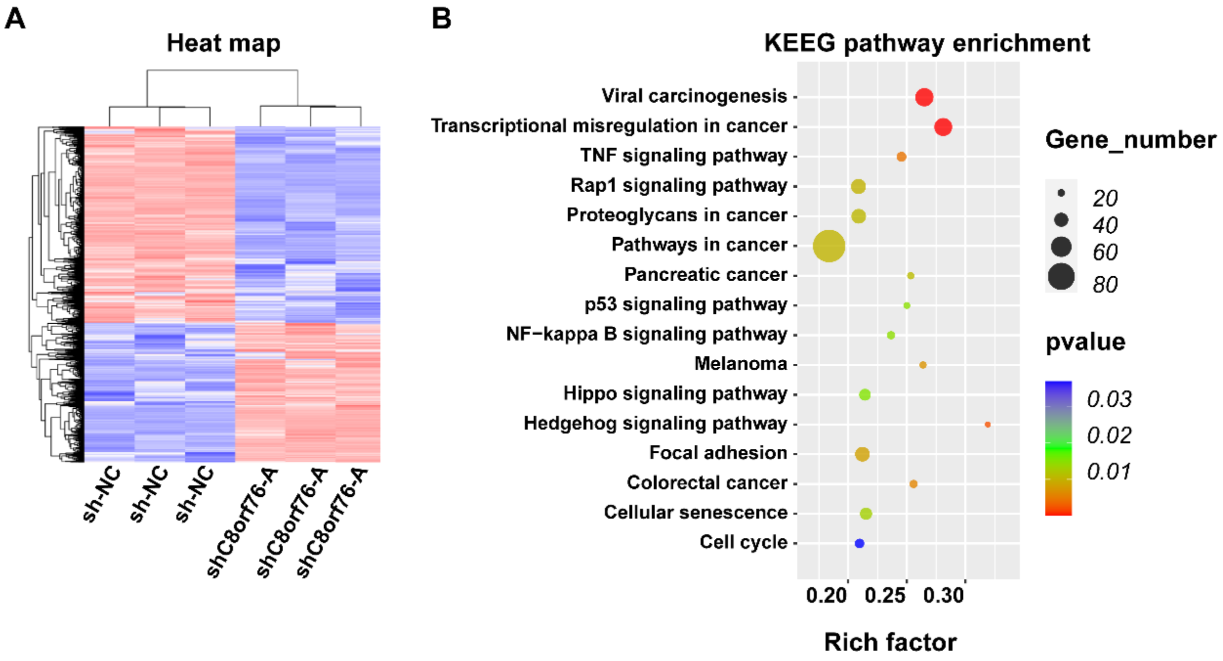

Figure S3. (**A**) The overall gene expression changes in HA22T cells with or without shC8orf76 infection, and upregulated genes presented in red while downregulated genes in blue (n=3). (**B**) The KEEG pathway enrichment analysis upon those changed genes with |log2FC|>1.

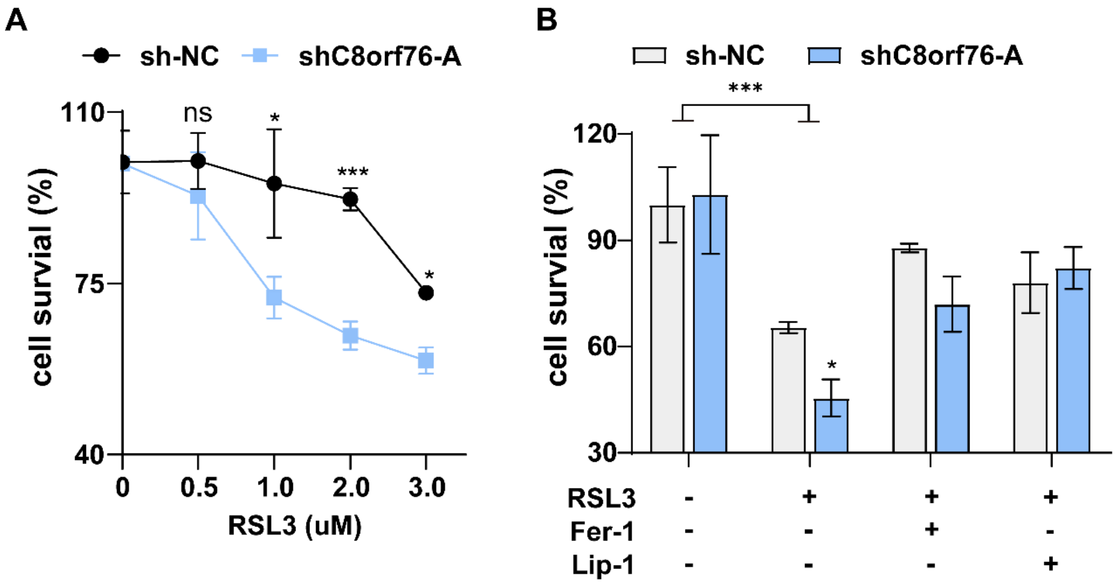

Figure S4. C8orf76 knockdown sensitizes HCC cells to GPX4 inhibitor-induced ferroptosis. (**A**) JHH7 cells were exposed to different doses of RSL3 for 24h and detected by MTS reagent. (**B**) The rescue effect of ferroptosis inhibitors on RSL3 treatment (2.5μM) was explored through MTS assay. Data was presented as mean+SD. * $p < 0.05$; ** $p < 0.01$; *** $p < 0.001$; ns, no significant.

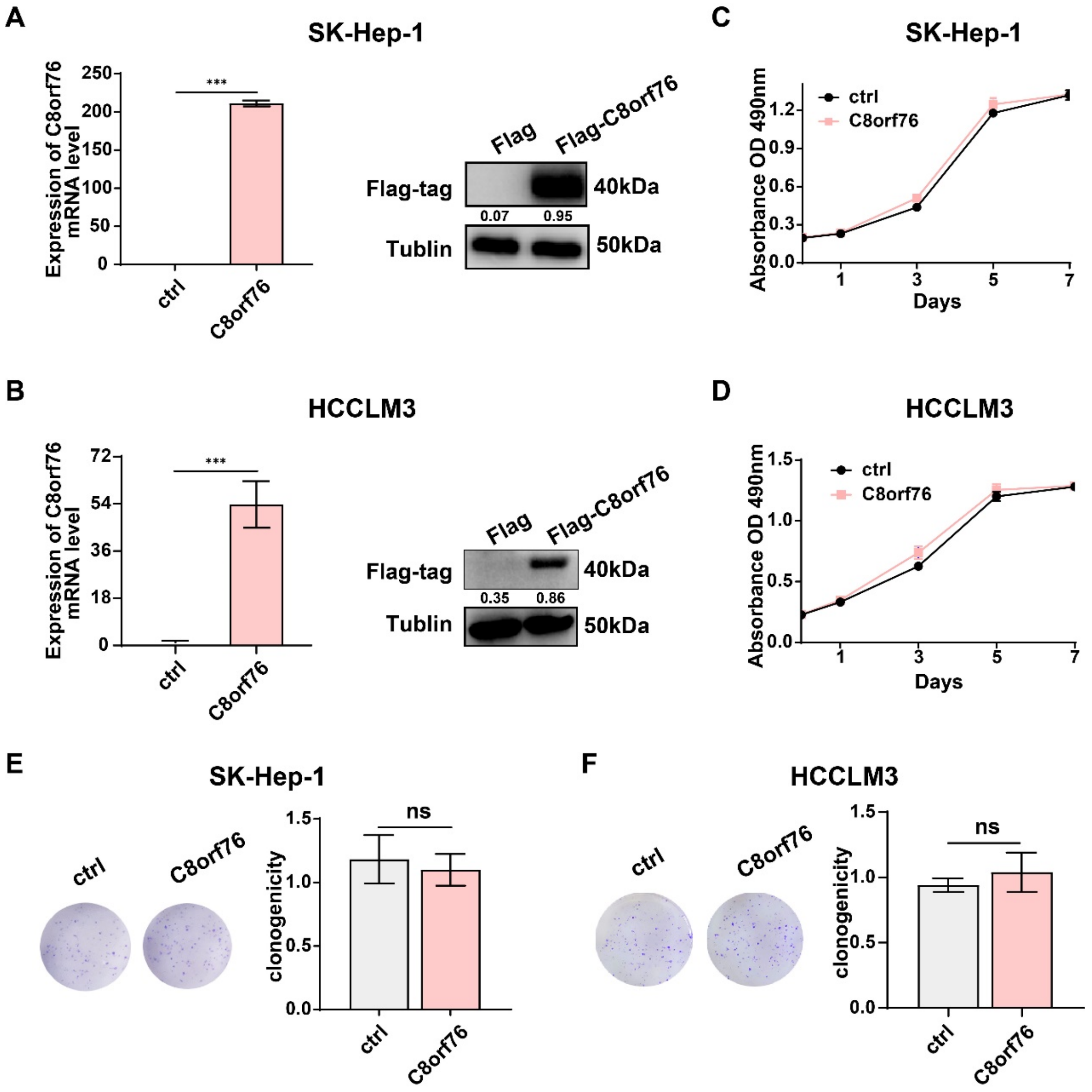

Figure S5. Analysis of cell growth in HCC upon C8orf76 overexpression. (**A**,**B**) The mRNA and protein levels of C8orf76 in SK-Hep-1 and HCCLM3 with ctrl or C8orf76 transfection. (**C**,**D**) MTS assay was used to measure HCC cell growth at indicated time while upregulating C8orf76 (n=3). (E-F) The colony formation of HCC cell lines with ctrl or C8orf76 transfection. Data was presented as mean+SD. * $p < 0.05$; ** $p < 0.01$; *** $p < 0.001$; ns, no significant.

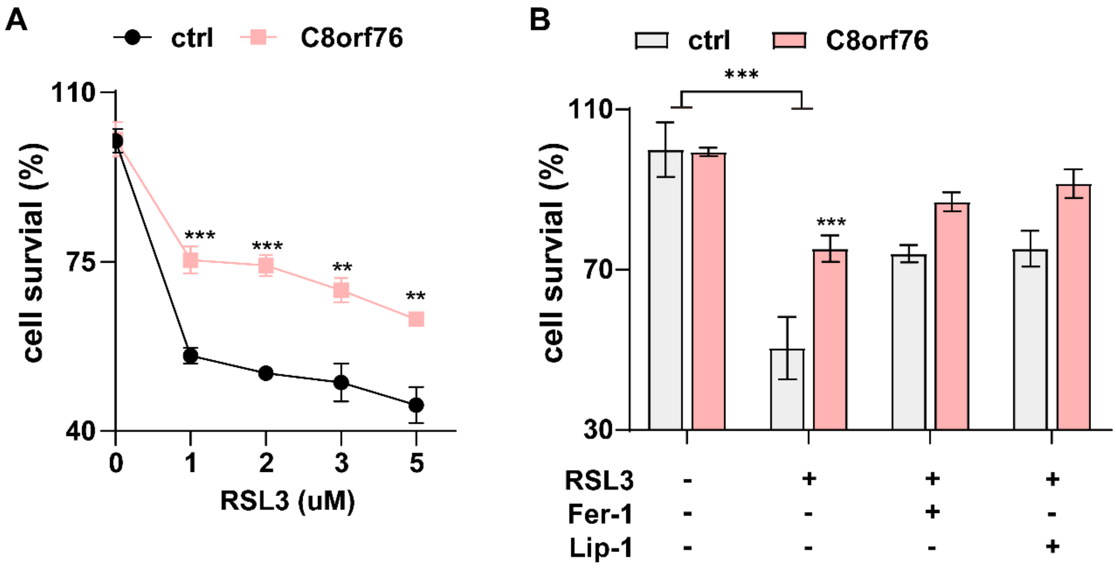

Figure S6. C8orf76 overexpression mediated ferroptosis resistance towards GPX4 inhibitor. (A) RSL3 treatment resulted in a dose-dependent inhibition of cell viability. Cell survival was measured by MTS. (B) MTS assay was performed to monitor the cell viability induced by RSL3 (2.5μM) combined with or without ferroptosis inhibitors (Fer-1, 1μM; Lip-1, 0.4μM). Data was presented as mean+SD. * $p < 0.05$; ** $p < 0.01$; *** $p < 0.001$; ns, no significant.

A

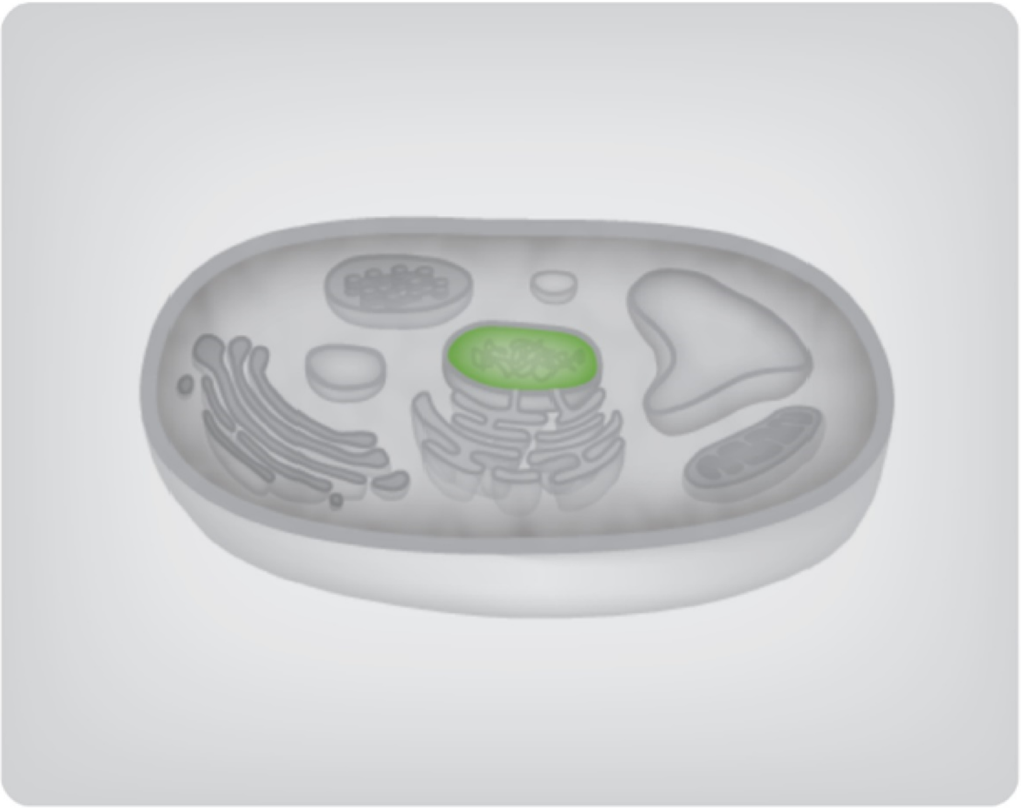

Figure S7. Online prediction of C8orf76 distribution in cells (PredictProtein).

Table S1. DNA sequences of primers used in this study.

Primer Name	Sequence (5'-3')
**RT-PCR**	
GAPDH-F	TTGGTATCGTGGAAGGACTCA
GAPDH-R	TGTCATCATATTTGGCAGGTT
C8orf76-F	TTATACGAACCAGGCTTCTGC
C8orf76-R	GCCAACACACTTCACCTCTG
SLC7A11-F	TTACCAGCTTTTGTACGAGTCT
SLC7A11-R	GTGAGCTTGCAAAAGGTTAAGA
**CHIP-qPCR**	
SLC7A11-F	GAGCTGAGTAATGCTGGAG
SLC7A11-R	AGCTCAGCTTCCTCATGG

Table S2. The sequence for the PGL3-SLC7A11 promoter construct.

Gene Name	Sequence (5'-3')
SLC7A11	GCTTTGTTCTCTAAAAAGCTTAGGTCAGTTGAG-<br>CAACAAGCTCCTC<br>CTGTTTTTTTCTTTTTTTAAAAAAAAGAGCTGAG-<br>TAATGCTGGAGGC<br>TTCTCATGTGGCTGATGCAAACCTGGAGAATTT-<br>GCATCATCATTTAG<br>CTGTAGTAAGTT-<br>GGTGTGACAGGCAGGCGCTTAAATACAAGCCCAT<br>GAGGAAGCTGAGCT
